# The Raid of the M.D.’s

**Published:** 1889-03

**Authors:** 


HALL'S
JOURNAL OF HEALTH

TRUTH DEMANDS NO SACRIFICE; ERROR CAN MAKE NONE.

VOL. 36. MARCH, 1889. No. 3.

### THE RAID OF THE M.D.'S.

The annual raid of the regularly diplomaed M.D. upon our several State Legislatures is now, for the most part, under full headway. The members of this exclusive order have persistently demanded lawful recognition of their right to treat the sick to the exclusion of all other persons, upon the ground of their superior qualifications, and they demand of the law-making powers the enforcement of this pretended right by legal enactment. Such a law would leave to the afflicted, or to his or her immediate relatives, no choice of remedies outside of the enforced prescriptions of some one of the recognized medical schools. It would, in fact, create a privileged order to whose ministrations every household would be subject at periods of deepest affliction, although in opposition to their views as to the necessities of the case, or the kind of treatment most likely to afford the requisite relief. No possible good has ever resulted from compulsory laws of this description. They are, indeed, akin to sumptuary laws which from time immemorial have failed of their purpose and are now almost wholly discouraged. How would a proposition be met which required all persons to purchase shoes or underwear of a certain make on the score of healthfulness. Such a law would be scarcely less reasonable than one which would require all diseased persons to take into their systems a particular class of drugs, administered by certain persons supposed to be skilled in their use. If there was any certainty in diagnosis or the efficacy of drugs in particular instances, it might be otherwise, but we have the authority of scores of the more eminent physicians the century has produced to the country. Since the experiments of Hippocrates, nearly five hundred years before the Christian era, the theory and practice of medicine have never demonstrated their claim to recognition as approaching anything like a science. While it is true that certain diseases have been named and classified as manifesting corresponding symptoms, for example, the several grades or descriptions of fever, it by no means follows that patients of dissimilar temperament and habits when suffering from the same general complaiut should undergo the same treatment. If drugs are resorted to they should be administered with a view to their special adaptation as remedial agents in every individual case, always keeping in view the state of the system produced by the patient's mode of life. What might benefit one might injure another. How many physicians trained in the schools are sufficiently discriminating to intelligently minister to the afflicted of all classes, ages and temperaments. Those who contend that poisonous remedies should scarcely ever be taken into the system, are, for the most part, non-graduates of any medical school, many of them being what are termed natural or magnetic healers, who, if they do not cure, certainly do not kill. It is this class that the M. D's seek to drive out of the field by Legislative aid. They want the whole field fenced in and left to themselves, so that should one die under their hands he may have the satisfaction of knowing he died by rule. Thus far our law-makers have rejected their demands, and, as we deem, wisely.

